# Rapid evolution of prey maintains predator diversity

**DOI:** 10.1371/journal.pone.0227111

**Published:** 2019-12-31

**Authors:** Akihiko Mougi

**Affiliations:** Institute of Agricultural and Life Sciences, Academic Assembly, Shimane University, Nishikawatsu-cho, Matsue, Japan; National Cheng Kung University, TAIWAN

## Abstract

Factors maintaining the populations of diverse species that share limited resources or prey remain important issues in ecology. In the present study, I propose that heritable intraspecific variation in prey, which facilitates natural selection, is a key to solve this issue. A mathematical model reveals that diverse genotypes in a prey promote the coexistence of multiple predator species. When two predators share a prey with multiple genotypes, evolution nearly selects the two prey genotypes. Through analysis, I establish a condition of coexistence of such multiple predator–one prey interaction with two genotypes. If each prey type has high defensive capacity against different predator species, stable coexistence is likely to occur. Particularly, interspecific variations of life-history parameters allow the coexistence equilibrium to be stable. In addition, rapid evolution in a prey allows more than two predator species to coexist. Furthermore, mutation tends to stabilize otherwise unstable systems. These results suggest that intraspecific variation in a prey plays a key role in the maintenance of diverse predator species by driving adaptive evolution.

## Introduction

Understanding the factors that facilitate coexistence among species is one of the most important issues in ecology [1,2]. According to the principle of competitive exclusion, the number of sympatric species competing for a common set of limited resources cannot surpass the amount of resources or prey species [3–7]. However, in nature, diverse competing species coexist [8]. Earlier theoretical studies proposed different mechanisms of coexistence [9]. Most of the proposed mechanisms are external factors of the system, such as selective predation by a shared top predator, temporal variability, or spatial heterogeneity [10–12]. Nevertheless, few mechanisms do not rely on external factors (e.g., internal non-equilibrium dynamics) [13] to explain coexistence. For example, some studies have shown that non-equilibrium dynamics in population densities alone are able to promote the coexistence of two consumers on a single resource based on relative non-linearity in the functional responses of two consumers [14,15]. Here, I propose an additional general internal factor, intraspecific diversity in prey, which facilitates adaptive evolution in prey.

Recent studies have started to explore the roles of intraspecific diversity or variation [16–21], which had been ignored in classical ecological dynamic models despite conspecific individuals varying in numerous traits, such as morphology, behavior, or physiology. Because the differences in such traits translate into differences in ecological features, such as anti-predator defenses [22] and resource utilization [23,24], they influence reproduction and/or survival (i.e., fitness). In addition, because most traits exhibit heritable variations at least to some extent [25,26], adaptive evolution in response to ecological interactions could occur [27]. Therefore, intraspecific variation could affect the classical ecological models via trait variation and changes in mean trait values in populations [23].

The evolution of interaction traits under the influence of interactions between or among species could occur on time scales similar to those of ecological population dynamics [28–33]. If evolution is rapid enough to influence population dynamics, feedback between ecological and evolutionary processes could considerably alter the population dynamics expected on the basis of the ecological population dynamics theory [34–39]. Such eco-evolutionary dynamics frameworks usually assume homogeneous populations at each time step, while overlooking the effects of intraspecific variation.

Recent studies have demonstrated that trait adaptation via intraspecific variation can promote multiple predator coexistence [40,41]. However, these studies usually track the continuous trait distribution, and rarely track the dynamics of individual phenotypes, and overlook the effects of individual responses of discrete phenotypes, such as polyphenism, in response to selection pressure.

Here, I have developed a population dynamics model in which multiple predators compete for a single prey with intraspecific variation. The prey comprises multiple genotypes with different predation rates for each predator and/or growth rate. Evolution is driven by a replicator equation, which is a general model describing natural selection [42,43]. In the ecological model, predators cannot exist without the evolution of the prey. Therefore, by controlling the genotype number in the prey (*N*_*G*_), evolution could occur. In the basal model, I assumed clonal prey populations or perfect inheritance of phenotypes. Although it is the simplest model of the evolution of discrete prey genotypes, the model makes it difficult to distinguish models of a single prey species and multiple prey species. To overcome this point, I also considered mutation between genotypes, which is a key to characterize within and among species. Using this eco-evolutionary dynamics model, I have demonstrated that evolution of a prey facilitates the coexistence of multiple competing predators, which suggests that intraspecific diversity in a prey sustains predator diversity by driving adaptive evolution.

## Methods

### Ecological dynamics

Consider the following population dynamics model of multiple predator–one prey interaction, in which the population of the prey species involves multiple genotypes:

$$\frac{dX}{dt}=\bar{w}X,$$ (1)

$$\frac{dY_{i}}{dt}=\left(g_{i}\sum_{j\in 1,\ldots ,N_{G}}a_{ij}f_{j}X-d_{i}\right)Y_{i},$$ (2)

where *X* and *Y*_*i*_ are prey and predator population sizes, respectively. *a*_*ij*_ is the consumption rate of the predator species *i* for prey genotype *j*, *g*_*i*_ (< 1) is the conversion efficiency linked to predator *i*’s birth rate based on prey consumption; and *d*_*i*_ is the death rate of the predator *i*. *N*_*G*_ represents the number of genotypes within the prey species, and *f*_*i*_(*i*∈1,…, *N*_*G*_) is the proportion of the genotypes in the population of the prey species (Σ_*i*∈1,…,*NG*_ *f*_*i*_ = 1).

$\bar{w}$ is the mean fitness averaged over genotypes within the population of prey species, represented by

$$\bar{w}=\sum_{i\in 1,\ldots ,N_{G}}f_{i}w_{i},$$ (3)

where *w*_*i*_ is the fitness of a genotype *i* of the prey species, represented by

$$w_{i}=r_{i}\left(1-\frac{X}{K}\right)-\sum_{j\in predators}a_{ji}Y_{j},$$ (4)

where *r*_*i*_ is the intrinsic growth rate in a genotype *i* of the prey species, and *K* is the carrying capacity of the prey.

### Evolutionary dynamics

I have modeled the dynamics of the proportions of genotypes within the population of the prey species *f*_*i*_ using a replicator equation [42,43] as follows:

$$\frac{df_{i}}{dt}=f_{i}(w_{i}-\bar{w}),$$ (5)

The differential Eqs (1), (2) and (5) describe the coupled ecological and evolutionary dynamics.

## Results

**Fig 1:**
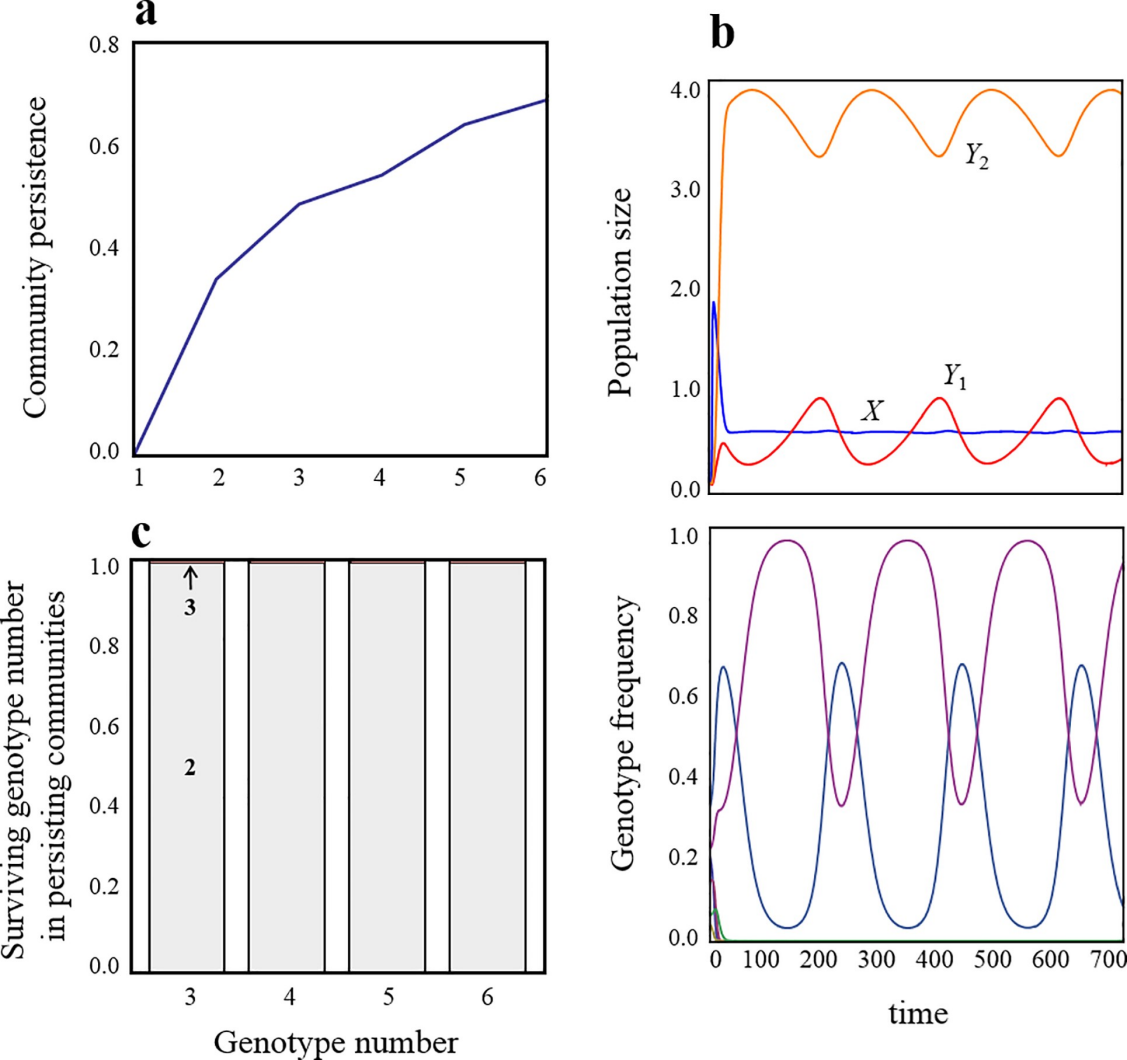
Eco-evolutionary dynamics and its consequences on coexistence: (a) Effects of genotype number on community persistence (see below). (b) Typical dynamics between population size and genotype frequency. (c) Surviving genotype number in persisting communities. Community persistence is the probability that all species persist during a sufficiently long period. It was calculated by measuring the frequencies of all co-occurring species (*X*, *Y*_*i*_ > 10^−5^ for all *i*) after sufficiently long periods (t = 5 × 10^3^ by which time community persistence reaches an asymptote) in 500 runs. In addition, surviving genotype number in persisting communities was calculated and the proportion of different numbers of surviving genotypes was plotted in (c) (2 or 3). In each iterated simulation, initial species abundances, genotype frequencies, and *a*_*ij*_ are randomly chosen from a uniform distribution, U(0, 1). Parameter values are *r*_*i*_ = 2.0, *K =* 2.0, *g*_*i*_ = 0.5, and *d*_*i*_ = 0.1. The parameters *a*_*ij*_ used in (b) are shown in S1 Text.

Consider an ecological community in which two predators compete for a single prey species. When the prey species comprises a homogeneous genotype (*N*_*G*_ = 1), evolution does not occur. In such a classical exploitative competition system with type 1 functional response of predators, the competing predators cannot coexist (Fig 1A). However, when the prey population is heterogeneous (*N*_*G*_ > 2), evolution occurs (Fig 1B), which qualitatively alters the ecological consequences. Evolution, therefore, allows competing predators to coexist. In addition, as the number of genotypes increases, the predators are more likely to coexist (Fig 1A). However, it is critical to note that at the evolutionary endpoint, the number of coexisting prey genotypes reduces, typically converging into two types (Fig 1B and 1C).

Here, I analyzed the simplest or most frequently observed system at the evolutionary equilibrium (*N*_*G*_ = 2). A mathematical analysis (S1 Text) reveals the condition under which the two predators can coexist (the coexistence equilibrium is feasible). First, each predator prefers different prey genotypes or each prey genotype has a high defensive ability against different predator species (e.g., *a*_11_ < *a*_12_, *a*_21_ > *a*_22_), and the offense ability of predators to each prey genotype is a trade-off relationship. More specialized defenses of each genotype against different predators also make the coexistence easier, which suggests that large differences in ecological characteristics of prey genotypes result in larger regions of parameter space where coexistence is possible. In contrast, even when predators prefer same prey genotype (e.g., *a*_11_ > *a*_12_, *a*_21_ > *a*_22_), coexistence is also possible if the preferred prey genotype grows faster than the non-preferred prey genotype.

**Fig 2:**
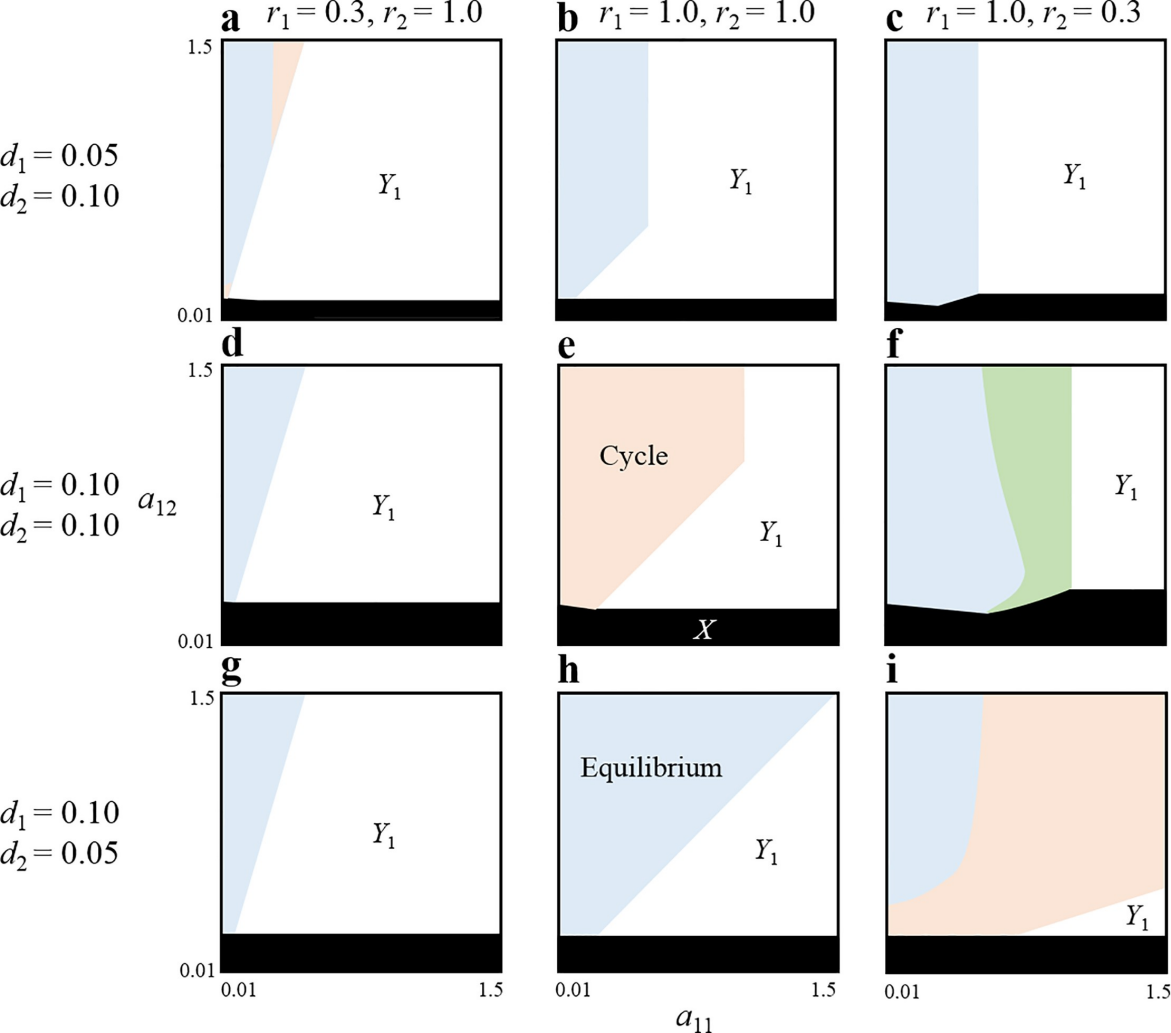
Relationship between the consumption rates and local stability of the equilibrium in two predator–one prey with two genotypes system: In orange regions, the non-trivial equilibrium is locally unstable and cycles occur. In blue regions, the equilibrium is locally stable and globally stable. In green region, the equilibrium is locally stable but globally unstable (a stable limit cycle occurs). In white regions, coexistence does not occur (*Y*_1_ exists and *Y*_2_ goes extinct). In black region, predators do not persist. Parameter values *r*_*i*_ and *d*_*i*_ are varied in each panel (a–i). Other parameter values are: *K =* 1.0, *g*_*i*_ = 0.5, *a*_21_ = 1.0, and *a*_12_ = 0.1.

Even if the equilibrium is feasible, the two prey genotypes and two predator species may not stably coexist because the equilibrium may not be stable. The local stability analysis revealed that coexistence is always unstable, and the dynamics demonstrate a stable limit cycle when life-history parameters excluding consumption rate (*a*_*ij*_) are symmetrical or same among predators and among prey genotypes (Fig 2E, S1 Fig, S1 Text). Stable coexistence is not possible even if the predators prefer to utilize different prey genotypes. However, once the symmetry assumption is relaxed, coexistence could become stable (Fig 2, S2 Fig). The asymmetries between the growth rates of prey genotypes and/or death rates of predator species facilitate stable coexistence. More specifically, a main prey type for a weak predator (with high death rate) needs to rapidly grow (Fig 2, S1 Text). This result supports the view that the intraspecific variation of various traits in prey plays a key role in facilitating species coexistence.

**Fig 3:**
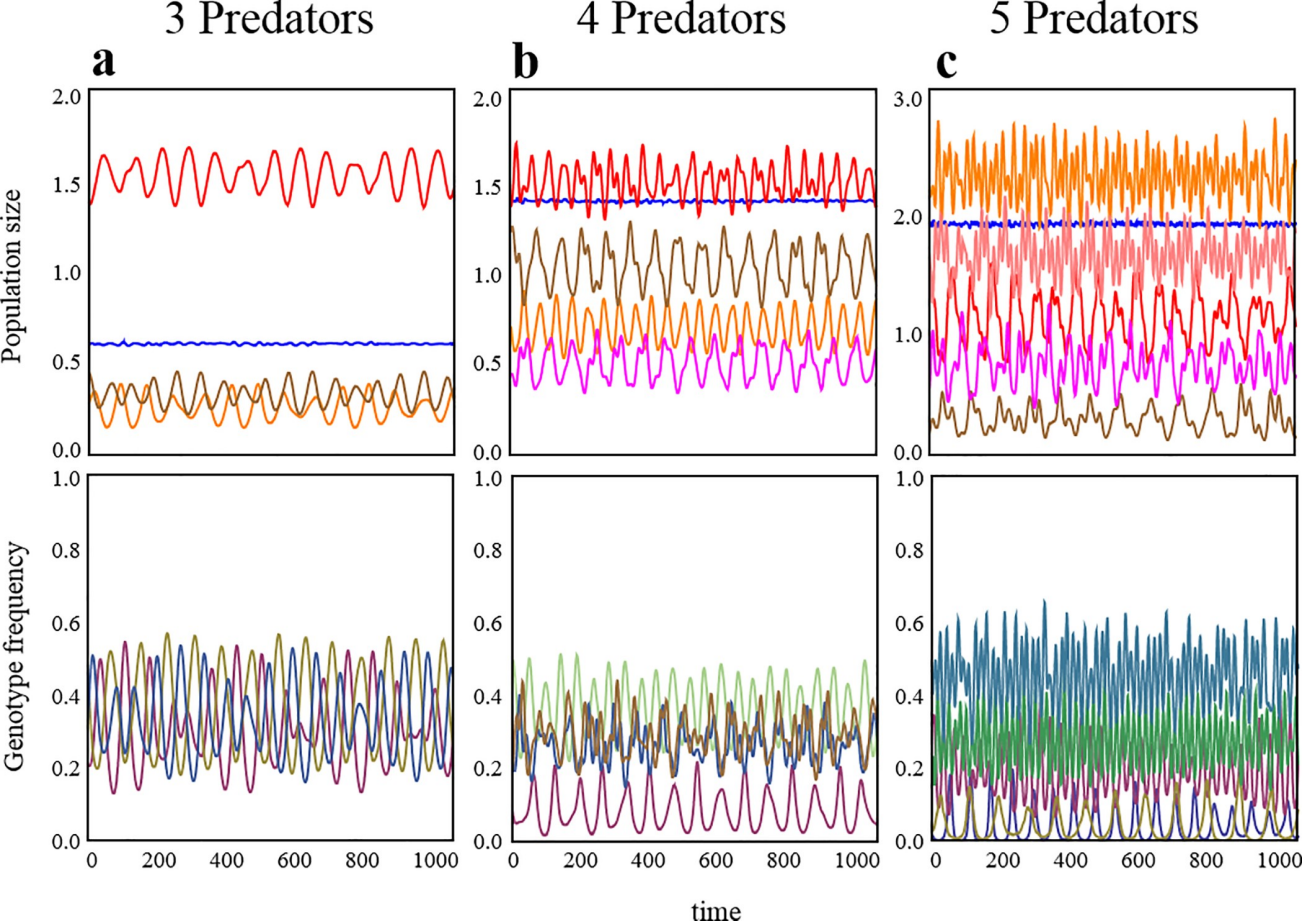
Examples of eco-evolutionary dynamics in predator systems with more than two predators: (a) Three predators coexist, (b) Four predators coexist, (c) Five predators coexist. In (a–c), initial genotype numbers are 6, 8, and 10, respectively. Parameters used in each case are shown in S1 Text.

Until now, for the purpose of simplicity, I have considered the coexistence problem of two competing predators, while leaving the question of whether intraspecific variation in prey driven evolution allows more than three multiple predators to coexist unaddressed. In Fig 3, I illustrate a case where more than three predators (3–5) competing for a single prey species can coexist. In addition, stable coexistence is possible as with two predator–one prey system (S2 Fig). In all cases, evolution selects the same number of prey genotypes with coexisting predators, similar to in the case of two predators. However, there is a limit to the number of predators that could be supported by a single prey. More diverse prey genotypes are required for the coexistence of diverse predators (Fig 1A, S3 Fig). More specifically, the diversity at the prey level needs to be at least as large as the one at higher trophic levels.

**Fig 4:**
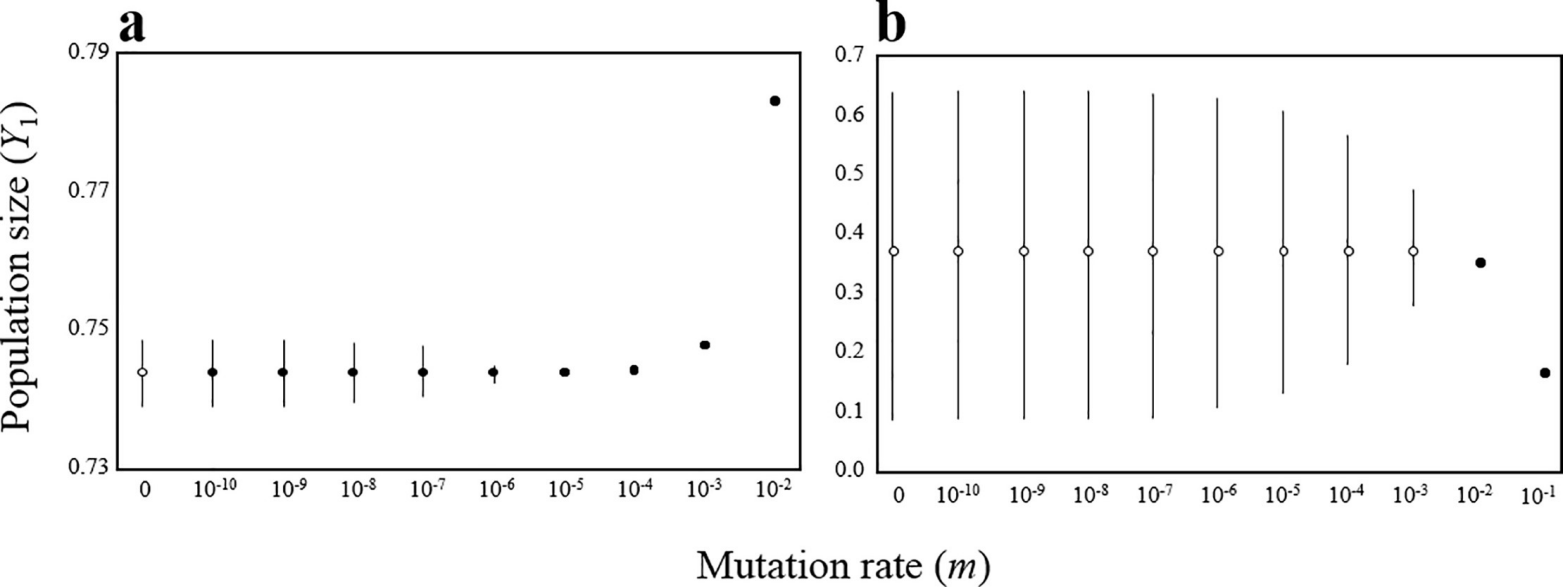
Population dynamics result in relation to the value of the mutation rate *m*. (a) *a*_11_ = 0.8, *a*_12_ = 1.0, *a*_21_ = 1.0, and *a*_22_ = 0.1 (other parameters are same as Fig 2E). (b) *a*_11_ = 1.2, *a*_12_ = 0.5, *a*_21_ = 1.0, and *a*_22_ = 0.1 (other parameters are same as Fig 2I). Open and closed circles indicate locally unstable and stable equilibrium, respectively. Bars indicate the oscillation ranges of population size *Y*_1_ (the results of *Y*_2_ have same tendency). Note that closed circles with bars are locally stable but globally unstable equilibrium. Equilibrium points are numerically obtained, and ranges of oscillations are obtained after sufficiently long periods (t = 10^7^).

It is clear that the above model is mathematically equivalent to a multiple predator–multiple prey model (S1 Text) because of asexual inheritance of genotypes. Here, I relaxed the strong assumption to consider a mutation between genotypes. For simplicity, I focused on the two predator–one prey with two genotypes system. Specifically, I added new terms of mutation into the r.h.s of Eq (5), *m*(1 − *f*_*i*_) − *mf*_*i*_, where *m* is the mutation rate. When *m* = 0, it is exactly the same with the above basal model without mutation. The analysis shows that mutation has two effects on stability of the system. First, it allows the locally unstable equilibrium to be locally or globally stable. Second, it can reduce the amplitudes of population cycles. The stabilization becomes stronger as mutation rate increases (Fig 4, S4 and S5 Figs). These suggest that the mutation plays a key role in stabilizing predator–prey systems.

## Discussion

Earlier works have contended that the coexistence of competing species sharing a limited resource is improbable contrary to observations in nature or in the field [8]. The present theory demonstrates that intraspecific trait diversity in a prey maintained by adaptive evolution of prey could sustain predator diversity. Higher intraspecific diversity in a prey could promote the coexistence of predator species. In addition, predators’ stable coexistence is probable when they exhibit differences in their preferences for the different prey genotypes or one prey genotype has a higher capacity to defend itself against different predators. More specifically, the different rates of predation in prey genotypes allow a limited cycle of fluctuating coexistence in each species. In addition, the effects of additional differences in parameters on the growth of prey, inter-genotypic competition, and predator survival facilitate stable coexistence. In addition, mutation allows unstable equilibrium to be stable. Furthermore, prey evolution allows more than two multiple predators to coexist. Intraspecific diversity in prey, via evolution, may be a key factor sustaining predator diversity.

It is not surprising that intraspecific variation in a single prey promotes the coexistence of the multiple predators. If prey genotypes are regarded as diverse resources, the present theory is essentially consistent with classical coexistence theory, which predicts that different species are limited by different resources [44]. However, a key point suggested by the results of present study is that natural ecosystems may have more abundant resources than previously thought and the coexistence of competing species is easier than expected. Classical models omitting intraspecific diversity may overestimate the difficulty in maintaining interspecific diversity. Overall, the results of the present study demonstrate that intraspecific diversity in prey could play a key role in sustaining predator diversity through adaptive evolution.

Recent theoretical studies support the present finding. Another standard model on evolutionary dynamics is quantitative genetics, which tracks the temporal changes in mean trait values of a population. Previous studies have shown that trait adaptive evolution based on quantitative genetics can promote supersaturated coexistence [40,41,45], i.e., where the number of consumers exceeds the number of potential resources. For example, a recent study showed that three predators are able to coexist on a single prey when the prey species is able to adapt its trait distribution, i.e., the mean and variance of the trait distribution, in response to altered selection pressure [41]. Hence, the present coexistence mechanism via intraspecific variation can be general without depending on modeling ways of evolution.

Genetic factors and mechanisms not considered in the present model could also contribute to the generation of intraspecific variations in traits. In the present model, replicator equation, one of the most simple models used to explain evolutionary dynamics of phenotypes or genotypes in a population without a detailed analysis of genetic effect [42,43], was used. Genetic architectures can prevent the loss of genotypes by natural selection [46,47], in turn, playing a role in the maintenance of variation. For example, a recent study [21] using a similar exploitative competition model in which a single locus with two alleles determines prey traits, prey nutritional quality, and predation rates, revealed that diploid prey could have stabilizing effect on community dynamics through a genetic storage effect contrary to haploid prey. More specifically, when heterozygotes are defended in a better manner against predators compared to homozygotes, fitness disadvantage in rare alleles could be mitigated by aiding the maintenance of the alleles, resulting in the stabilization of the system and maintenance of genetic polymorphism [21]. This study suggests that a genetic detail does not destroy the present coexistence mechanism. Conversely, because other enormous genetic details will be conceivable depending on the types of evolving traits, further studies will be necessary to show the robustness of the coexistence mechanism. Although in the present model the number of prey genotypes tended to converge with the number of predators to create an equilibrium [31], genetic architecture would maintain prey genotypes more than predicted even without mutation. Since individual phenotypes are created by various qualitative and quantitative traits and their combinations, real populations should exhibit much greater intraspecific variation, suggesting that the present coexistence mechanism is likely to apply in nature.

The present study has important implications for biodiversity conservation. Loss of intraspecific diversity would be considered as loss of genetic diversity, which would reduce the capacity of a species to evolve in response to environmental changes and cause inbreeding depression [48]. The loss of intraspecific diversity could also have implications beyond the reduction of survival ability in a focal species. Since such reductions in intraspecific diversity could lead to a reduction in resource niches supporting predators, which, in turn, could result in the loss of species diversity.

## Supporting information

S1 Text Mathematical analyses. (DOCX)

S1 Code Mathematica code for reproducing figures. (DOCX)

S1 Fig The relationship between the consumption rates and local stability of the equilibrium in a two predator–one prey with two genotypes system: In orange regions, the non-trivial equilibrium is locally unstable and cycles occur. In white regions, coexistence does not occur (*Y*_1_ or *Y*_2_ is feasible). In black regions, predators do not persist. Parameter values are *r*_*i*_ *=* 1.0, *K =* 1.0, *g*_*i*_ *=* 0.5, and *d*_*i*_ = 0.1.
(TIF)

S2 Fig Examples of eco-evolutionary dynamics toward stable equilibrium: (a) Two predator–one prey with two genotypes system: (b) Three predator–one prey with six genotypes. Note that the three genotypes survived in (b). Prey population size are represented by blue (other colors are predators). In (a), *K =* 1.0, *r*_1_ *=* 0.3, *r*_2_ *=* 1.0, *g*_*i*_ = 0.5, *a*_11_ = 0.1, *a*_12_ = 1.0, *a*_21_ = 1.0, *a*_22_ = 0.1, *d*_1_ = 0.1, and *d*_2_ = 0.05. In (b), *K =* 1.0, *r*_1_ *=* 1.36554, *r*_2_ *=* 0.717423, *r*_3_ *=* 1.94401, *r*_4_ *=* 1.58014, *r*_5_ *=* 0.880044, *r*_6_ *=* 1.63521, *g*_*i*_ = 0.5, *d*_1_ = 0.0945787, *d*_2_ = 0.0950382, and *d*_3_ = 0.151273 (see S1 Text for values of *a*_*ij*_).
(TIF)

S3 Fig Effects of genotype number to community persistence in multiple predator cases: In each different line, the systems have different number of predators. Parameters are same as those used in Fig 1A.
(TIF)

S4 Fig Population dynamics result in relation to the value of the mutation rate *m*: (a) *a*_11_ = 0.9, *a*_12_ = 1.0, *a*_21_ = 1.0, and *a*_22_ = 0.1 (other parameters are same as Fig 2E). (b) *a*_11_ = 1.2, *a*_12_ = 1.0, *a*_21_ = 1.0, and *a*_22_ = 0.1 (other parameters are same as Fig 2I). Other information is same as Fig 4.
(TIF)

S5 Fig Effects of mutation rate to population oscillation in relation to *K*: I assumed a11 = 0.9, *a*_12_ = 1.0, *a*_21_ = 1.0, and *a*_22_ = 0.8. Other parameters are same as Fig 2E.
(TIF)

S6 Fig A typical dynamics of two predator–one prey with two genotypes system in the limit without self-regulation (no carrying capacity): Parameter values are: *r_i_* = 1.0, *g_i_* = 0.5, *d_i_* = 0.1, *a*_11_ = 0.5, *a*_12_ = 1.2, *a*_21_ = 1.0, and *a*_22_ = 0.3. (TIF)

## References

[1] Gause GF, The struggle for existence (New York, NY: Hafner Publishing Company, 1934). 10.1126/science.79.2036.16-a 17821472

[2] Hutchinson GE, The paradox of the plankton. *Am Nat* 95, 137–145 (1961).

[3] Volterra V, Variations and fluctuations of the number of individuals in animal species living together. *J Cons Cons Int Explor Mer* 3, 3–51 (1928).

[4] MacArthur R, Levins R, Competition, habitat selection, and character displacement in a patchy environment. *Proc Natl Acad Sci USA* 51, 1207–1210 (1964). 10.1073/pnas.51.6.1207 14215645 PMC300237

[5] Rescigno A, Richardson IW, On the competitive exclusion principle. *Bull Math Biophys* 27, 85–89 (1965). 10.1007/BF02477264 5884143

[6] Levins R, Evolution in changing environments: some theoretical explorations (Princeton Univ. Press, Princeton, NJ, 1968).

[7] Levin SA, Community equilibria and stability, and an extension of the competitive exclusion principle. *Am Nat* 104, 413–423 (1970).

[8] Hutchinson GE, Homage to santa rosalia or why are there so many kinds of animals? *Am Nat* 93, 145–159 (1959).

[9] Chesson P, Mechanisms of maintenance of species diversity. *Annu Rev Ecol Syst* 31, 343–366 (2000).

[10] Levins R, Coexistence in variable environment. *Am Nat* 114, 765–783 (1979).

[11] Chase JM, Abrams PA, Grover JP, Diehl S, Chesson P, Holt RD, et al The interaction between predation and competition: a review and synthesis. *Ecol Lett* 5, 302–315 (2002).

[12] Amarasekare P, Competitive coexistence in spatially structured environments: a synthesis. *Ecol Lett* 6, 1109–1122 (2003).

[13] Huisman J, Weissing FJ, Biodiversity of plankton by species oscillations and chaos. *Nature* 402, 407–410 (1999).

[14] Abrams PA, Holt RD, The impact of consumer-resource cycles on the coexistence of competing consumers. *Theor Popul Biol* 62, 281–295 (2002). 10.1006/tpbi.2002.1614 12408947

[15] Armstrong RA, Mcgehee R, Competitive-Exclusion. *Am Nat* 115, 151–170 (1980).

[16] Haigh J, Maynard Smith J, Can there be more predators than prey? *Theor Popul Biol* 3, 290–299 (1972). 10.1016/0040-5809(72)90005-6 4667088

[17] Bolnick DI, Amarasekare P, Araújo MS, Bürger R, Levine JM, Novak M, et al Why intraspecific trait variation matters in community ecology. *Trend Ecol Evol* 26, 183–192 (2011). 10.1016/j.tree.2011.01.009 PMC3088364 21367482

[18] Schreiber SJ, Bürger R, Bolnick DI, The community effects of phenotypic and genetic variation within a predator population. *Ecology* 92, 1582–1593 (2011). 10.1890/10-2071.1 21905425

[19] Menden-Deuer S, Rowlett J, Many ways to stay in the game: individual variability maintains high biodiversity in planktonic microorganisms. J Royal Soc Inter 11, 20140031 (2014). 10.1098/rsif.2014.0031 PMC4006243 24647904

[20] Menden-Deuer S, Rowlett J, The theory of games and microbe ecology. *Theor Ecol* 12, 1–15 (2019).

[21] Schreiber SJ, Patel S, Terhorst C, Evolution as a coexistence mechanism: Does genetic architecture matter? *Am Nat* 191, 407–420 (2018).

[22] Duffy MA, Ecological consequences of intraspecific variation in lake Daphnia. *Freshw Biol* 55, 995–1004 (2010).

[23] Bolnick DI, Svanbäck R, Fordyce JA, Yang LH, Davis JM, Hulsey CD, et al The ecology of individuals: incidence and implications of individual specialization. *Am Nat* 161, 1–28 (2003). 10.1086/343878 12650459

[24] Lankau RA, Strauss SY, Mutual feedbacks maintain both genetic and species diversity in a plant community. *Science* 317, 1561–1563 (2007). 10.1126/science.1147455 17872447

[25] Roff DA, *Evolutionary Quantitative Genetics* (Chapman and Hall, 1997).

[26] Lynch M, Walsh B, *Genetics and Analysis of Quantitative Traits* (Sinauer Associates, Inc, 1998).

[27] Fussmann GF, Loreau M, Abrams PA, Eco-evolutionary dynamics of communities and ecosystems. *Funct Ecol* 21, 465–477 (2007).

[28] Yoshida T, Jones LE, Ellner SP, Fussmann GF, Hairston NG Jr, Rapid evolution drives ecological dynamics in a predator-prey system. *Nature* 424, 303–306 (2003). 10.1038/nature01767 12867979

[29] Hairston NG Jr, Ellner SP, Geber M, Yoshida T, Fox JE, Rapid evolution and the convergence of ecological and evolutionary time. *Ecol Lett* 8, 1114–1127 (2005).

[30] Bailey JK, Schweitzer JA, Úbeda F, Koricheva J, LeRoy CJ, Madritch MD, et al From genes to ecosystems: a synthesis of the effects of plant genetic factors across levels of organization. Phil Trans Roy Soc B: B*iol Sci* 364, 1607–1616 (2009). 10.1098/rstb.2008.0336 PMC2690499 19414474

[31] Jones LE, Becks L, Ellner SP, Hairston NG Jr, Yoshida T, Fussmann G, Rapid contemporary evolution and clonal food web dynamics. Phil Trans Roy Soc B: Biol *Sci* 364, 1579–1591 (2009). 10.1098/rstb.2009.0004 PMC2690500 19414472

[32] Palkovacs EP, Marshall MC, Lamphere BA, Lynch BR, Weese DJ, Fraser DF, et al Experimental evaluation of evolution and coevolution as agents of ecosystem change in Trinidadian streams. Phil Trans Roy Soc B: B*iol Sci* 364, 1617–1628 (2009). 10.1098/rstb.2009.0016 PMC2690507 19414475

[33] Post DM, Palkovacs EP, Eco-evolutionary feedbacks in community and ecosystem ecology: interactions between the ecological theatre and the evolutionary play. Phil Trans Roy Soc B: Bi*ol Sci* 364, 1629–1640 (2009). 10.1098/rstb.2009.0012 PMC2690506 19414476

[34] Abrams PA, The evolution of predator-prey interactions: theory and evidence. *Annu Rev Ecol Syst* 31, 79–105 (2000).

[35] Cortez MH, Ellner SP, Understanding rapid evolution in predator-prey interactions using the theory of fast-slow dynamical systems. *Am Nat* 176, E109–E127 (2010). 10.1086/656485 20863225

[36] Mougi A, Iwasa Y, Evolution towards oscillation or stability in a predator-prey system. *Proc R Soc B* 277, 3163–3171 (2010). 10.1098/rspb.2010.0691 20504808 PMC2982064

[37] Mougi Iwasa Y, Unique coevolutionary dynamics in a predator-prey system. *J Theor Biol* 277, 83–89 (2011). 10.1016/j.jtbi.2011.02.015 21354181

[38] Mougi A, Predator prey coevolution driven by size selective predation can cause anti-synchronized and cryptic population dynamics. *Theor Popul Biol* 81,113–118 (2012). 10.1016/j.tpb.2011.12.005 22212374

[39] Cortez MH, Weitz JS, Coevolution can reverse predator-prey cycles. *Proc Nat Acad Sci USA* 111, 7486–7491 (2014). 10.1073/pnas.1317693111 24799689 PMC4034221

[40] Klauschies T, Vasseur DA, Gaedke U, Trait adaptation promotes species coexistence in diverse predator and prey communities. *Ecol Evol* 6, 4141–4159 (2016). 10.1002/ece3.2172 27516870 PMC4972238

[41] Klauschies T, Coutinho RM, Gaedke U, A beta distribution-based moment closure enhances the reliability of trait-based aggregate models for natural populations and communities. Ecol Model 381, 46–77 (2018).

[42] Taylor PD, Jonker LB, Evolutionary stable strategies and game dynamics. *Math Biosci* 40,145–156 (1978).

[43] Schuster P, Sigmund K, Replicator dynamics. *J Theor Biol* 100, 533–538 (1983).

[44] Tilman D, Resources: a graphical-mechanistic approach to competition and predation. *Am Nat* 116, 363–393 (1980).

[45] Abrams PA, The prerequisites for and likelihood of generalist-specialist coexistence. *Am Nat* 167, 329–342 (2006). 10.1086/499382 16673342

[46] Gillespie JH, Pleiotropic overdominance and the maintenance of genetic variation in polygenic characters. *Genetics* 107, 321–330 (1984). 6735172 10.1093/genetics/107.2.321 PMC1202325

[47] Gillespie JH, Turelli M, Genotype-environment interactions and the maintenance of polygenic variation. *Genetics* 121, 129–138 (1989). 17246488 10.1093/genetics/121.1.129 PMC1203595

[48] Frankhan R, Genetics and extinction. *Biol Conserv* 126, 131–140 (2005).

