# Supplementary material for: Rapid evolution of prey maintains predator diversity
Source: PLoS One. 2019 Dec 31;14(12):e0227111. doi: 10.1371/journal.pone.0227111 (PMC6938327; doi:10.1371/journal.pone.0227111)
Supplement: S1 Text — (DOCX) [file pone.0227111.s001.docx]

**Supplementary Information**

**Appendix**

***Local stability analysis***

The local stability of the system described by equations (1) and (4) was performed by linearizing the dynamics near the nontrivial equilibrium. Stability was judged by whether the characteristic equation of their Jacobian matrix satisfied the Routh–Hurwitz criteria.

The Jacobian matrix (not shown because of too complex) was calculated under the equilibrium:

*X*^*^ = *A*/*g*_1_*g*_2_*B*, (S-1a)

*Y*_1_^*^ = *C*_1_(*A* – *g*_1_*g*_2_*KB*)/*g*_1_*g*_2_*KB*^2^, (S-1b)

*Y*_2_^*^ = *C*_2_(*A* – *g*_1_*g*_2_*KB*)/*g*_1_*g*_2_*KB*^2^, (S-1c)

*f_X_^*^* = (*g*_2_*d*_1_*a*_22_ – *g*_1_*d*_2_*a*_12_)/*A*, (S-1d)

where *A = g*_1_*d*_2_(*a*_11_ − *a*_12_) + *g*_2_*d*_1_(*a*_22_ – *a*_21_), *B =* *a*_11_*a*_22_ − *a*_12_*a*_21_, *C*_1_ *= r*_2_*a*_21_ – *r*_1_*a*_22_ and *C*_2_ *= r*_1_*a*_12_ – *r*_2_*a*_11_. A feasible condition of non-trivial equilibrium, *X*^*^ > 0, *Y*_1_^*^ > 0, *Y*_2_^*^ > 0 and 0 < *f_X_^*^*< 1, is summarised as below,

$\frac{a_{12}}{a_{11}}>\theta_{1}>\frac{a_{22}}{a_{21}}$, (S-2a)

$\frac{a_{11}}{a_{21}}<\theta_{2}<\frac{a_{12}}{a_{22}}$, (S-2b)

$0<\theta_{3}<K$ (S-2c)

where *θ*_1_ = *r*_2_/*r*_1_, *θ*_2_ = (*g*_2_/*d*_2_)/(*g*_1_/*d*_1_), and *θ*_3_ = *g*_1_*g*_2_*A*/*B* (= [{(*a*_11_−*a*_12_)/(*g*_2_/*d*_2_)}+{(*a*_22_− *a*_21_)/(*g*_1_/*d*_1_)}]/(*a*_12_*a*_21_−*a*_11_*a*_22_). In the above case, I arbitrarily chose a condition *C_i_* > 0, however it may be the opposite one (i.e., *C_i_* < 0). Note that since the two cases are essentially identical, the argument never change. First, consider a case where parameters except for *a_ij_* are symmetrical. In this case, the inequalities (S-2a and 2b) requires *a*_22_ < *a*_12_ > *a*_11_ < *a*_21_ > *a*_22_. This implies that each predator prefers different prey genotypes or each prey genotype has a high defensive ability against different predator species, and the offense ability of predators to each prey genotype is in the trade-off relationship. Next, consider a case where parameters except for *a_ij_* are asymmetrical. Then, even when predators prefer a same prey genotype (*a*_11_ > *a*_12_, *a*_21_ > *a*_22_), coexistence is possible, if the preferred prey genotype grows faster than non-preferred prey genotype (*r*_1_ > *r*_2_). In addition, the inequality (S-2c) shows that more specialized defenses of each genotype against different predators decrease the lower limit of *K* and broaden the stability region.

The characteristic equation for determining the eigenvalues is$\lambda^{4}+w_{1}\lambda^{3}+w_{2}\lambda^{2}+w_{3}\lambda+w_{4}=0.$The equilibrium point is locally stable if *w*_1_, *w*_3_, *w*_4_ > 0 and $w_{1}\left(w_{2}w_{3}-w_{1}w_{4}\right)-w_{3}^{2}>0,$ according to the Routh–Hurwitz criteria. Unfortunately, the mathematical analysis of the local stability in the full model is difficult, because of too complex. For analytical tractability, consider three special cases. In addition, for the simplicity, I assume *g_i_* = *g* in the following analysis.

**(1) Symmetrical parameters except for *a_ij_***

The first one is *r_i_* = *r*, *d_i_* = *d*. Then, the feasible condition of the non-trivial equilibrium is as below,

*a*_11_ < *a*_12_, (S-3a)

*a*_11_ < *a*_21_, (S-3b)

*a*_22_ < *a*_12_, (S-3c)

*a*_22_ < *a*_21_, (S-3d)

$\left(\frac{a_{12}-a_{11}+a_{21}-a_{22}}{a_{12}a_{21}-a_{11}a_{22}}\right)\frac{d}{g}<K$ (S-3e)

Under the equilibrium, I obtain the coefficients of characteristic equation, *w_i_* (*i* =1,..., 4). Then, I found that a necessary condition for stability, $w_{1}\left(w_{2}w_{3}-w_{1}w_{4}\right)-w_{3}^{2}>0$, is never held ($w_{1}\left(w_{2}w_{3}-w_{1}w_{4}\right)-w_{3}^{2}=0$). Hence, in this special case, the equilibrium is always unstable. This suggests that the stable coexistence is not possible even if the predators prefer to utilize different prey genotypes.

**(2) Equal predation rates of two predators to different prey genotypes**

Finally, consider the second special case, *a*_12_ = *a*_21_ = *a* and *a*_11_ = *a*_22_ = *a*’. Here, I assume *a* > *a*’ (the argument is essentially the same even if the condition is reversed). Then, the feasible condition of the non-trivial equilibrium is as below,

$\frac{1}{\alpha}>v_{1}>\alpha$, (S-4a)

$\frac{1}{\alpha}>v_{2}>\alpha$, (S-4b)

$K>\frac{d_{1}+d_{2}}{g\left(a+a'\right)}$ (S-4c)

where *v*_1_ = *d*_2_/*d*_1_, *v*_2_ = *r*_2_/*r*_1_ and *α* = *a*’/*a.*

Under the equilibrium, I obtain the coefficients of characteristic equation, *w_i_* (*i* = 1,..., 4). By using feasible equilibrium condition, the stability condition is the following:

$\frac{v_{1}+v_{2}}{1+v_{1}v_{2}}>\alpha,$ (S-5a)

$\left(\frac{v_{1}-v_{2}}{1-v_{1}v_{2}}\right)^{2}>\alpha^{2}$ (S-5b)

First, these inequalities are likely to be held when *α* is small, suggesting that stability requires more specialised predation of two predators to different prey genotypes. In addition, those conditions are likely to be held when *v*_1_ > 1 > *v*_2_ or *v*_1_ < 1 < *v*_2_, suggesting that a main prey type of a weak predator (with high death rate) needs to rapidly grow.

The above two special cases suggest that stability requires both niche division in predators and asymmetry of life-history parameters among predators and prey genotypes. Full model analysis performed by numerically calculating eigenvalues of Jacobian matrix supports this mathematical result (see main text and Fig. 2).

**(3) Very large *K* (no self-regulation)**

Next, consider the third special case with very large *K* (=∞). Then, the feasible condition of the non-trivial equilibrium is as below,

$\frac{a_{12}}{a_{11}}>\theta_{1}>\frac{a_{22}}{a_{21}}$, (S-6a)

$\frac{a_{11}}{a_{21}}<\theta_{2}<\frac{a_{12}}{a_{22}}$, (S-6b)

Under the equilibrium, I obtain the coefficients of characteristic equation, *w_i_* (*i* =1,..., 4). Then, I found that a necessary condition for stability, *w*_1_ > 0, is always not held (*w*_1_ = 0). Hence, in this special case, the equilibrium is always unstable, suggesting that self-regulation is required for the stability. In this system, the dynamics shows a chaotic behaviour (Fig. S4).

Full model analysis was performed by numerically calculating eigenvalues of Jacobian matrix (see main text).

***Parameter values used in Fig. 1b*, *3 and S2b.***

Encounter rates of the predator *i* with respect to the prey with genotype *j* (*j*$\in$1,…,6), *a_ij_*, are given in matrix *A*. Different columns represent different predator species, and different rows represent different prey genotypes.

In Fig. 1b, *A* is given by:

*A =*$\left(\begin{matrix} 0.5266033824664371 & 0.8348848701823657 \\ 0.1535041754551505 & 0.6405915811590421 \\ 0.8523666639151404 & 0.9375987876548548 \\ 0.15726183650439074 & 0.4338872034834129 \\ 0.19707587545850913 & 0.35411727848786323 \\ 0.3809732633509293 & 0.3259784677952655 \end{matrix}\right)$

In Fig. 3a, *A* is given by:

$$\left(\begin{matrix} 0.6785611993250191 & 0.6402739133032618 & 0.8583428104476374 \\ 0.6791813578079147 & 0.21713498325052005 & 0.9090701262019374 \\ 0.5732905087764983 & 0.9520186813833944 & 0.8350322719323728 \\ 0.6657846214991483 & 0.7619018873588328 & 0.6913704434586327 \\ 0.7358820856257324 & 0.7517091561486695 & 0.23705952280073062 \\ 0.6237406667963779 & 0.6594920369435222 & 0.8918979489136332 \end{matrix}\right)$$

The other parameters are: *r_i_* = 2.0, *K* = 2.0, *g_i_* = 0.5, and *d_i_* = 0.2.

In Fig. 3b, *A* is given by:

$$\left(\begin{matrix} 0.8345970115295815 & 0.7901409118741789 & 0.9809183639778309 & 0.32147462493406964 \\ 0.2567261055630081 & 0.6453269230670877 & 0.6950524535823144 & 0.10910469308057424 \\ 0.5469494636681145 & 0.2127962392323699 & 0.44147215422584374 & 0.3722704423804464 \\ 0.9183385503244423 & 0.3347262399832518 & 0.15153874142112034 & 0.8533069036853065 \\ 0.17384744919416917 & 0.3715471607549279 & 0.6221441159625942 & 0.8931733256974166 \\ 0.20081242181306758 & 0.5898824405380028 & 0.7811877310248032 & 0.28532054445934696 \\ 0.5710384955395866 & 0.6954219161467352 & 0.17003535163915895 & 0.18554522822770503 \\ 0.9827571223641451 & 0.9867823760601682 & 0.4027332065864748 & 0.913397088243705 \end{matrix}\right)$$

The other parameters are: *r_i_* = 3.0, *K* = 3.0, *g_i_* = 0.5, and *d_i_* = 0.3.

In Fig. 3c, *A* is given by:

$$\left(\begin{matrix} 0.8911913297149079 & 0.389941648632792 & 0.6528320199505286 & 0.14442206795319446 & 0.2071869534013535 \\ 0.4888203106639839 & 0.24880262617766102 & 0.8593690426940175 & 0.7997371384762644 & 0.3465446784859352 \\ 0.28147100887710175 & 0.4907365673653088 & 0.9874143305951029 & 0.20467889298085007 & 0.399419659459332 \\ 0.5087526964014253 & 0.21261541219498592 & 0.2206953165810135 & 0.1629833462070106 & 0.7931701153429311 \\ 0.17941871496675432 & 0.38184736077602066 & 0.5509124706839361 & 0.7259877955804974 & 0.9772850737862593 \\ 0.872781571177536 & 0.7716552759838835 & 0.9063667414852834 & 0.6562539956698921 & 0.7714413809507861 \\ 0.12247968484006222 & 0.8137863846412865 & 0.31312358068463264 & 0.7060567174373047 & 0.65260070632452 \\ 0.6076059413099049 & 0.5703953810126279 & 0.143634595860068 & 0.5411169239509948 & 0.5600706726030678 \\ 0.3018279353352187 & 0.6071197285557568 & 0.2780012356324395 & 0.46210145031981487 & 0.24213785723304282 \\ 0.6196291627396691 & 0.6643742957373464 & 0.2624975804533136 & 0.7443664912088628 & 0.6346693716347824 \end{matrix}\right)$$

The other parameters are: *r_i_* = 4.5, *K* = 4.5, *g_i_* = 0.5, and *d_i_* = 0.4.

In Fig. S2b, *A* is given by:

$$\left(\begin{matrix} 0.6505348939240481 & 0.6338177172291437 & 0.14918248496447584 \\ 0.9003735160396626 & 0.7061170226913178 & 0.16220589875096725 \\ 0.6542659936715887 & 0.37206430171104987 & 0.9162005773635324 \\ 0.7267440095517985 & 0.1769608617697226 & 0.7812907080397231 \\ 0.5580515156069938 & 0.36101141880900367 & 0.14108737596573206 \\ 0.25059714964506985 & 0.5528747294004046 & 0.7923091093458077 \end{matrix}\right)$$

***Consistency with the explicit genotype dynamics model***

I can show that the present model is exactly same with explicit genotype dynamics model (or multiple prey species model) as below.

First, I obtain mean fitness $\bar{w}=\frac{dX/dt}{X}$ by deforming the equation (1a) in the text. By substituting the mean fitness into the equation (4) in the text, I have

$\frac{df_{i}}{dt}=f_{i}(w_{i}-\frac{dX/dt}{X}),$ (S-7)

By multiplying both sides of the above equation (S-7) by *X*, I have

$\frac{df_{i}}{dt}\cdot X=f_{i}Xw_{i}-f_{i}\frac{dX}{dt},$ (S-8)

By moving the second term in the right hand side of (S-8) to the left hand side, I have

$\frac{df_{i}}{dt}\cdot X+\frac{dX}{dt}f_{i}=f_{i}Xw_{i}$ (S-9)

Since the left hand side of (S-9) equals to d*f_i_X*/dt, equation (S-9) becomes to

$\frac{df_{i}X}{dt}=f_{i}Xw_{i}$ (S-10)

By defining *f_i_X* = *X_i_* (where *X_i_* is the population size of each genotype *i* in prey species), finally I have

$\frac{dX_{i}}{dt}=X_{i}w_{i}$ (S-11)
