# Supplementary material for: Rapid evolution of prey maintains predator diversity
Source: PLoS One. 2019 Dec 31;14(12):e0227111. doi: 10.1371/journal.pone.0227111 (PMC6938327; doi:10.1371/journal.pone.0227111)
Supplement: S1 Code — (DOCX) [file pone.0227111.s002.docx]

(*program for Fig.1a*)

Ng=2;

g=0.5;

d=0.1;

r=2;

a=1;

K=2;

gener=5000;

listdata={};

Do[

wx=Table[r (1-X[t]/K)-a RandomReal[]Y1[t]-a RandomReal[]Y2[t],{Ng}];

alis=Table[Table[wx[[j,i]][[1]],{i,2,3}],{j,1,Ng}];

flis=Table[f[i][t],{i,1,Ng-1}];

f[Ng][t]=1-Total[flis];

flis=AppendTo[flis,f[Ng][t]];

flis1=Table[f[i][t],{i,1,Ng-1}];

predgrow=-g Transpose[alis flis]X[t];

predfitness=Table[Total[predgrow[[i]]]-d,{i,1,2}];

meanfitness=Total[wx flis];

repli=flis1 Table[wx[[i]]-meanfitness,{i,1,Ng-1}];

repeq=Table[f[i]'[t]repli[[i]],{i,1,Ng-1}];

initialf=Table[RandomReal[],{Ng}];

initialf=initialf/Total[initialf];

Delete[initialf,Ng];

initialf=Delete[initialf,Ng];

initialf=Table[f[i][0]==initialf[[i]],{i,1,Ng-1}];

eqlis={X'[t]X[t]meanfitness,

Y1'[t]predfitness[[1]] Y1[t],

Y2'[t]predfitness[[2]] Y2[t],

repeq,

X[0]0.1,Y1[0]0.1,Y2[0]0.1,initialf};

eqlis=Flatten[eqlis];

fvar=Table[f[i],{i,1,Ng-1}];

var=Flatten[{X,Y1,Y2,fvar}];

s=NDSolve[eqlis,var,{t,0, gener},MaxStepsInfinity,MaxStepSize1,PrecisionGoalAutomatic,AccuracyGoalAutomatic];

listlast=Flatten[Table[Evaluate[{X[t],Y1[t],Y2[t]}/.s],{t,gener,gener}]];

listpersist={};

Do[If[listlast[[i]]>10^-5^,AppendTo[listpersist,listlast[[i]]]],{i,1,3}];

listfra=Flatten[Table[Evaluate[flis/.s],{t,gener,gener}]];

Do[

If[listfra[[i]]>0.99999listfra[[i]]<10^-5^,listfra[[i]]=1,listfra[[i]]=0],{i,1,Ng}];

AppendTo[listdata,{Length[listpersist],Count[listfra,0]}],{500}];

Count[Transpose[listdata][[1]],3]/500//N

(*program for Fig.1c*)

coeposi=Flatten[Position[Transpose[listdata][[1]],3]];

lisfr=Table[Transpose[listdata][[2]][[coeposi[[i]]]],{i,Length[coeposi]}];

Count[lisfr,2]

Count[lisfr,3]

Count[lisfr,4]

Count[lisfr,0]

(*profram for Fig. 1b*)

Ng=2;

g=0.5;

d=0.1;

r=2;

a=1;

K=2;

gener=5000;

wx=Table[r RandomReal[{0,4}] (1-X[t]/K)-a RandomReal[{0,1}]Y1[t]-a RandomReal[{0,1}]Y2[t],{Ng}];

alis=Table[Table[wx[[j,i]][[1]],{i,2,3}],{j,1,Ng}];

flis=Table[f[i][t],{i,1,Ng-1}];

f[Ng][t]=1-Total[flis];

flis=AppendTo[flis,f[Ng][t]];

flis1=Table[f[i][t],{i,1,Ng-1}];

predgrow=-g Transpose[alis flis]X[t];

predfitness=Table[Total[predgrow[[i]]]-d RandomReal[{0,0.2}],{i,1,2}];

meanfitness=Total[wx flis];

repli=flis1 Table[wx[[i]]-meanfitness,{i,1,Ng-1}];

repeq=Table[f[i]'[t]repli[[i]],{i,1,Ng-1}];

initialf=Table[RandomReal[],{Ng}];

initialf=initialf/Total[initialf];

Delete[initialf,Ng];

initialf=Delete[initialf,Ng];

initialf=Table[f[i][0]==initialf[[i]],{i,1,Ng-1}];

eqlis={X'[t]X[t]meanfitness,

Y1'[t]predfitness[[1]] Y1[t],

Y2'[t]predfitness[[2]] Y2[t],

repeq,

X[0]0.1,Y1[0]0.1,Y2[0]0.1,initialf};

eqlis=Flatten[eqlis];

fvar=Table[f[i],{i,1,Ng-1}];

var=Flatten[{X,Y1,Y2,fvar}];

s=NDSolve[eqlis,var,{t,0, gener},MaxStepsInfinity,MaxStepSize0.25,PrecisionGoalAutomatic,AccuracyGoalAutomatic];

Plot[Evaluate[{X[t],Y1[t],Y2[t]}/.s],{t,0,gener},PlotRange{0,5},PlotStyle{Blue,Red,Orange}]

Plot[Evaluate[flis/.s],{t,0,gener},PlotRange{0,1}]

(*Fig. 2*)

(*local stability of non-trivial equilibrium*)

(*note that trivial equilibria are analytically determined*)

r1=1;r2=1;

g1=g2=0.5;

d1=0.1;d2=0.1;K=1;

a21=1;a22=0.1;

wx1=r1 (1-X/K)-a11 Y1-a21 Y2;

wx2=r2 (1-X/K)-a12 Y1-a22 Y2;

wy1=g1 (a11 fx X+a12 (1-fx) X)-d1;

wy2=g2 (a21 fx X+a22 (1-fx) X)-d2;

Wx=wx1 fx+wx2 (1-fx);

Fx=wx1-(wx1 fx+wx2 (1-fx));

A11=D[Wx X,X];

A12=D[Wx X,Y1];

A13=D[Wx X,Y2];

A14=D[Wx X,fx];

A21=D[wy1 Y1,X];

A22=D[wy1 Y1,Y1];

A23=D[wy1 Y1,Y2];

A24=D[wy1 Y1,fx];

A31=D[wy2 Y2,X];

A32=D[wy2 Y2,Y1];

A33=D[wy2 Y2,Y2];

A34=D[wy2 Y2,fx];

A41=D[Fx fx,X];

A42=D[Fx fx,Y1];

A43=D[Fx fx,Y2];

A44=D[Fx fx,fx];

listlist={};

Do[

list={};

Do[

X=(-a11 d2 g1+a12 d2 g1+a21 d1 g2-a22 d1 g2)/(a12 a21 g1 g2-a11 a22 g1 g2);

Y1=(((-a21+a22) d1 g2+a12 g1 (-d2+a21 g2 K)+a11 g1 (d2-a22 g2 K)) (-a22 r1+a21 r2))/((a12 a21-a11 a22)^2^ g1 g2 K);Y2=(((-a21+a22) d1 g2+a12 g1 (-d2+a21 g2 K)+a11 g1 (d2-a22 g2 K)) (a12 r1-a11 r2))/((a12 a21-a11 a22)^2^ g1 g2 K);fx=(a12 d2 g1-a22 d1 g2)/((-a11+a12) d2 g1+(a21-a22) d1 g2);

J={{A11,A12,A13,A14},{A21,A22,A23,A24},{A31,A32,A33,A34},{A41,A42,A43,A44}};

If[X>0Y1>0Y2>0fx>0fx<1,

If[Max[Re[Eigenvalues[J]]]<0,listdumy=5,listdumy=4],

listdumy=1];

AppendTo[list,listdumy],{a11,0.01001,1.5,0.0101}];

AppendTo[listlist,list],{a12,0.01,1.501,0.01}];

ListContourPlot[listlist,InterpolationOrder0,Contours5]

(*Fig. 3*)

(*note that parameter values of "Encounter rates" was used in those of Supplemental files*)

(*Fig. 3a*)

Ng=6;

g=0.5;

d=0.2;

r=2;

a=1;

K=2;

gener=7000;

wx=Table[r (1-X[t]/K)-a RandomReal[{0.1,1}]Y1[t]-a RandomReal[{0.1,1}]Y2[t]-a RandomReal[{0.1,1}]Y3[t],{Ng}];

alis=Table[Table[wx[[j,i]][[1]],{i,2,4}],{j,1,Ng}];

flis=Table[f[i][t],{i,1,Ng-1}];

f[Ng][t]=1-Total[flis];

flis=AppendTo[flis,f[Ng][t]];

flis1=Table[f[i][t],{i,1,Ng-1}];

predgrow=-g Transpose[alis flis]X[t];

predfitness=Table[Total[predgrow[[i]]]-d ,{i,1,3}];

meanfitness=Total[wx flis];

repli=flis1 Table[wx[[i]]-meanfitness,{i,1,Ng-1}];

repeq=Table[f[i]'[t]repli[[i]],{i,1,Ng-1}];

initialf=Table[RandomReal[],{Ng}];

initialf=initialf/Total[initialf];

Delete[initialf,Ng];

initialf=Delete[initialf,Ng];

initialf=Table[f[i][0]==initialf[[i]],{i,1,Ng-1}];

eqlis={X'[t]X[t]meanfitness,

Y1'[t]predfitness[[1]] Y1[t],

Y2'[t]predfitness[[2]] Y2[t],

Y3'[t]predfitness[[3]] Y3[t],

repeq,

X[0]0.1,Y1[0]0.1,Y2[0]0.1,Y3[0]0.1,initialf};

eqlis=Flatten[eqlis];

fvar=Table[f[i],{i,1,Ng-1}];

var=Flatten[{X,Y1,Y2,Y3,fvar}];

s=NDSolve[eqlis,var,{t,0, gener},MaxStepsInfinity,MaxStepSize0.3,PrecisionGoalAutomatic,AccuracyGoalAutomatic];

Plot[Evaluate[{X[t],Y1[t],Y2[t],Y3[t]}/.s],{t,0,gener},PlotRange{0,5},PlotStyle{Blue,Red,Yellow,Brown}]

Plot[Evaluate[flis/.s],{t,0,gener},PlotRange{0,1}]

(*Fig. 3b*)

Ng=8;

g=0.5;

d=0.3;

r=3;

a=1;

K=3;

gener=5000;

wx=Table[r (1-X[t]/K)-a RandomReal[{0.1,1}]Y1[t]-a RandomReal[{0.1,1}]Y2[t]-a RandomReal[{0.1,1}]Y3[t]-a RandomReal[{0.1,1}]Y4[t],{Ng}];

alis=Table[Table[wx[[j,i]][[1]],{i,2,5}],{j,1,Ng}];

flis=Table[f[i][t],{i,1,Ng-1}];

f[Ng][t]=1-Total[flis];

flis=AppendTo[flis,f[Ng][t]];

flis1=Table[f[i][t],{i,1,Ng-1}];

predgrow=-g Transpose[alis flis]X[t];

predfitness=Table[Total[predgrow[[i]]]-d ,{i,1,4}];

meanfitness=Total[wx flis];

repli=flis1 Table[wx[[i]]-meanfitness,{i,1,Ng-1}];

repeq=Table[f[i]'[t]repli[[i]],{i,1,Ng-1}];

initialf=Table[RandomReal[],{Ng}];

initialf=initialf/Total[initialf];

Delete[initialf,Ng];

initialf=Delete[initialf,Ng];

initialf=Table[f[i][0]==initialf[[i]],{i,1,Ng-1}];

eqlis={X'[t]X[t]meanfitness,

Y1'[t]predfitness[[1]] Y1[t],

Y2'[t]predfitness[[2]] Y2[t],

Y3'[t]predfitness[[3]] Y3[t],

Y4'[t]predfitness[[4]] Y4[t],

repeq,

X[0]0.1,Y1[0]0.1,Y2[0]0.1,Y3[0]0.1,Y4[0]0.1,initialf};

eqlis=Flatten[eqlis];

fvar=Table[f[i],{i,1,Ng-1}];

var=Flatten[{X,Y1,Y2,Y3,Y4,fvar}];

s=NDSolve[eqlis,var,{t,0, gener},MaxStepsInfinity,MaxStepSize0.3,PrecisionGoalAutomatic,AccuracyGoalAutomatic];

Plot[Evaluate[{X[t],Y1[t],Y2[t],Y3[t],Y4[t]}/.s],{t,0,gener},PlotRange{0,4},PlotStyle{Blue,Red,Orange,Brown,Green}]

Plot[Evaluate[flis/.s],{t,0,gener},PlotRange{0,1}]

(*Fig.3c*)

Ng=10;

g=0.5;

d=0.4;

r=4.5;

a=1;

K=4.5;

gener=2000;

wx=Table[r (1-X[t]/K)-a RandomReal[{0.1,1}]Y1[t]-a RandomReal[{0.1,1}]Y2[t]-a RandomReal[{0.1,1}]Y3[t]-a RandomReal[{0.1,1}]Y4[t]-a RandomReal[{0.1,1}]Y5[t],{Ng}];

alis=Table[Table[wx[[j,i]][[1]],{i,2,6}],{j,1,Ng}];

flis=Table[f[i][t],{i,1,Ng-1}];

f[Ng][t]=1-Total[flis];

flis=AppendTo[flis,f[Ng][t]];

flis1=Table[f[i][t],{i,1,Ng-1}];

predgrow=-g Transpose[alis flis]X[t];

predfitness=Table[Total[predgrow[[i]]]-d ,{i,1,5}];

meanfitness=Total[wx flis];

repli=flis1 Table[wx[[i]]-meanfitness,{i,1,Ng-1}];

repeq=Table[f[i]'[t]repli[[i]],{i,1,Ng-1}];

initialf=Table[RandomReal[],{Ng}];

initialf=initialf/Total[initialf];

Delete[initialf,Ng];

initialf=Delete[initialf,Ng];

initialf=Table[f[i][0]==initialf[[i]],{i,1,Ng-1}];

eqlis={X'[t]X[t]meanfitness,

Y1'[t]predfitness[[1]] Y1[t],

Y2'[t]predfitness[[2]] Y2[t],

Y3'[t]predfitness[[3]] Y3[t],

Y4'[t]predfitness[[4]] Y4[t],

Y5'[t]predfitness[[5]] Y5[t],

repeq,

X[0]0.1,Y1[0]0.1,Y2[0]0.1,Y3[0]0.1,Y4[0]0.1,Y5[0]0.1,initialf};

eqlis=Flatten[eqlis];

fvar=Table[f[i],{i,1,Ng-1}];

var=Flatten[{X,Y1,Y2,Y3,Y4,Y5,fvar}];

s=NDSolve[eqlis,var,{t,0, gener},MaxStepsInfinity,MaxStepSizeInfinity,PrecisionGoalAutomatic,AccuracyGoalAutomatic];

Plot[Evaluate[{X[t],Y1[t],Y2[t],Y3[t],Y4[t],Y5[t]}/.s],{t,0,gener},PlotRange{0,5},PlotStyle{Blue,Red,Purple,Brown,Green,Yellow}]

Plot[Evaluate[flis/.s],{t,0,gener},PlotRange{0,1}]

(*Fig.4*)

r1=1;r2=1;

a11=0.9;a12=1;

a21=1;a22=0.1;

g1=0.5;g2=0.5;

d1=0.1;d2=0.1;

K=1;gener=10000000;

list={};

Do[

wx1=r1 (1-X[t]/K)-a11 Y1[t]-a21 Y2[t];

wx2=r2 (1-X[t]/K)-a12 Y1[t]-a22 Y2[t];

wy1=g1 (a11 fx[t] X[t]+a12 (1-fx[t]) X[t])-d1;

wy2=g2 (a21 fx[t] X[t]+a22 (1-fx[t]) X[t])-d2;

s=NDSolve[{X'[t]X[t](wx1 fx[t]+wx2 (1-fx[t])),

Y1'[t]wy1 Y1[t],

Y2'[t]wy2 Y2[t],

fx'[t] fx[t] (wx1-(wx1 fx[t]+wx2 (1-fx[t])))+q (1-fx[t])-q fx[t],

X[0]0.1,Y1[0]0.1,Y2[0]0.1,fx[0]0.2},{X,Y1,Y2,fx},{t,0,gener},MaxStepsInfinity,MaxStepSizeInfinity];

AppendTo[list,{Max[Table[Evaluate[Y1[t]/.s],{t,gener-9000000,gener}]],Min[Table[Evaluate[Y1[t]/.s],{t,gener-9000000,gener}]]}],{q,0,0,1}];

Flatten[list]

(*max and min in each value of q(mutation rate) are obtained *)

Y1=((a11 d2 g1-1.` a12 d2 g1-1.` a21 d1 g2+a22 d1 g2+a12 a21 g1 g2 K-1.` a11 a22 g1 g2 K) (-1.` a22 r1+a21 r2))/((a12 a21-1.` a11 a22)^2^ g1 g2 K);

(*equilibrium in q(mutation rate) is obtained *)
