# Supplementary figures and images for: Rapid evolution of prey maintains predator diversity
Source: PLoS One. 2019 Dec 31;14(12):e0227111. doi: 10.1371/journal.pone.0227111 (PMC6938327; doi:10.1371/journal.pone.0227111)

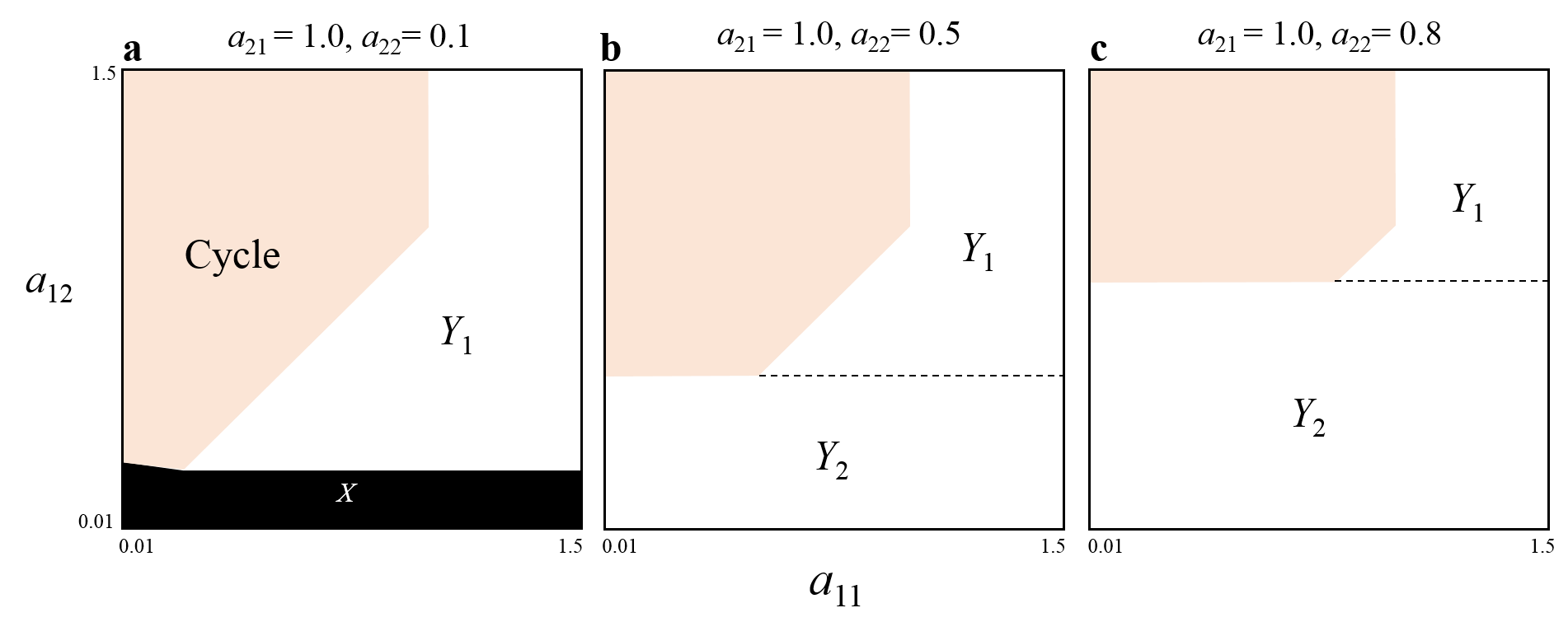

Supplement: S1 Fig — In white regions, coexistence does not occur (Y1 or Y2 is feasible). In black regions, predators do not persist. Parameter values are ri = 1.0, K = 1.0, gi = 0.5, and di = 0.1. (TIF) [file pone.0227111.s003.tif]

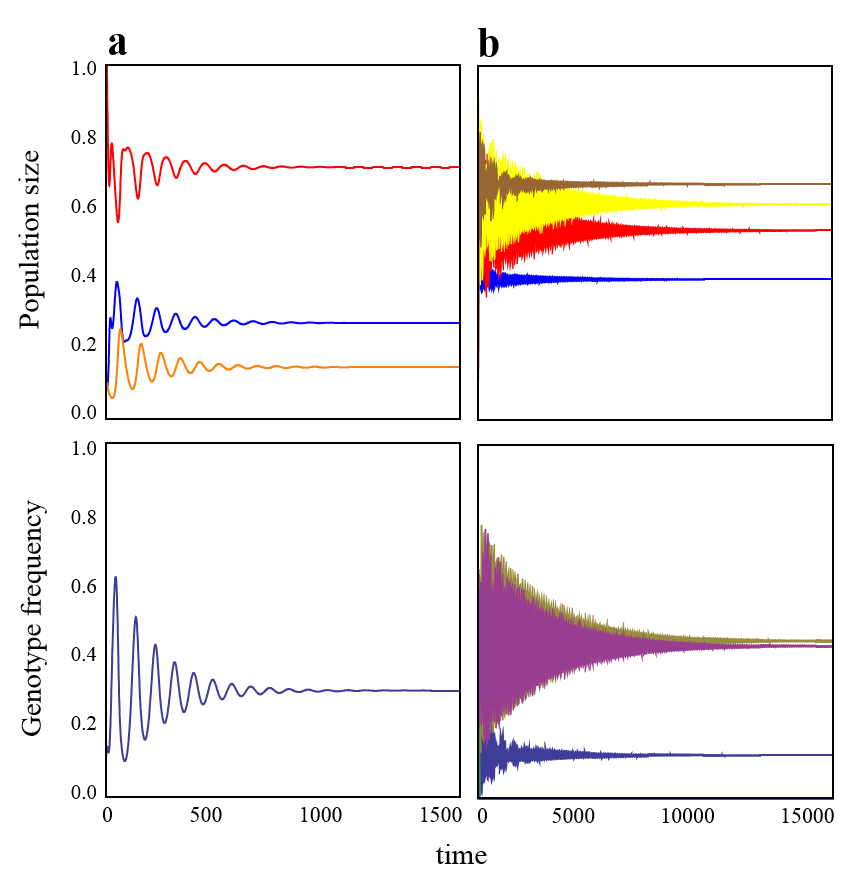

Supplement: S2 Fig — Examples of eco-evolutionary dynamics toward stable equilibrium: (a) Two predator–one prey with two genotypes system: (b) Three predator–one prey with six genotypes. Note that the three genotypes survived in (b). Prey population size are represented by blue (other colors are predators). In (a), K = 1.0, r1 = 0.3, r2 = 1.0, gi = 0.5, a11 = 0.1, a12 = 1.0, a21 = 1.0, a22 = 0.1, d1 = 0.1, and d2 = 0.05. In (b), K = 1.0, r1 = 1.36554, r2 = 0.717423, r3 = 1.94401, r4 = 1.58014, r5 = 0.880044, r6 = 1.63521, gi = 0.5, d1 = 0.0945787, d2 = 0.0950382, and d3 = 0.151273 (see S1 Text for values of aij). (TIF) [file pone.0227111.s004.tif]

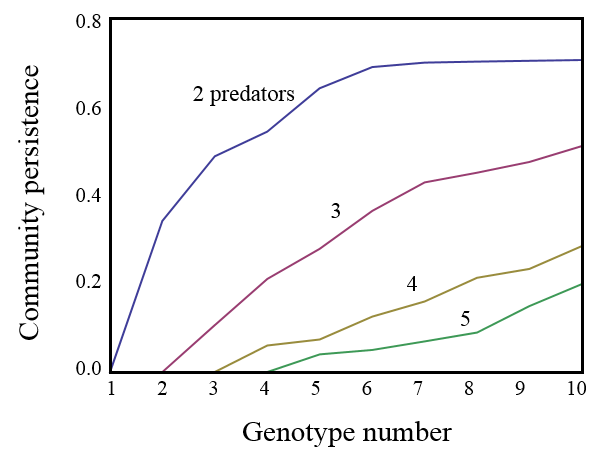

Supplement: S3 Fig — Parameters are same as those used in Fig 1A. (TIF) [file pone.0227111.s005.tif]

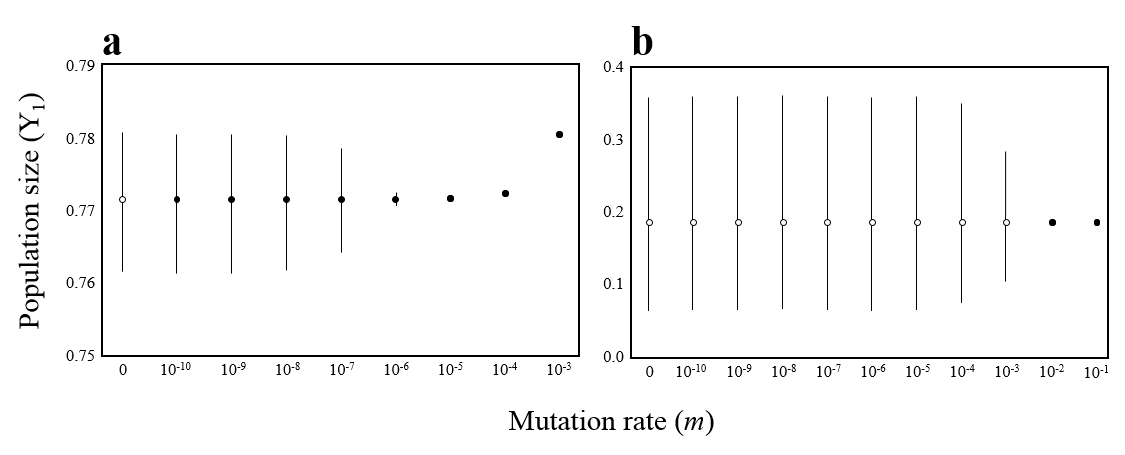

Supplement: S4 Fig — Population dynamics result in relation to the value of the mutation rate m: (a) a11 = 0.9, a12 = 1.0, a21 = 1.0, and a22 = 0.1 (other parameters are same as Fig 2E). (b) a11 = 1.2, a12 = 1.0, a21 = 1.0, and a22 = 0.1 (other parameters are same as Fig 2I). Other information is same as Fig 4. (TIF) [file pone.0227111.s006.tif]

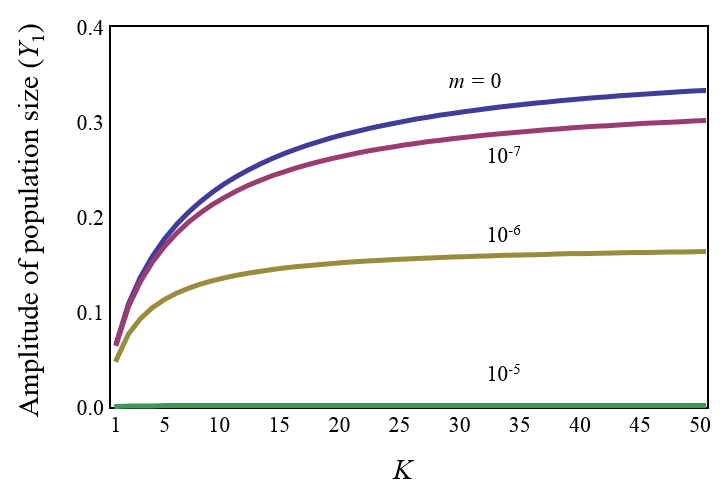

Supplement: S5 Fig — Other parameters are same as Fig 2E. (TIF) [file pone.0227111.s007.tif]

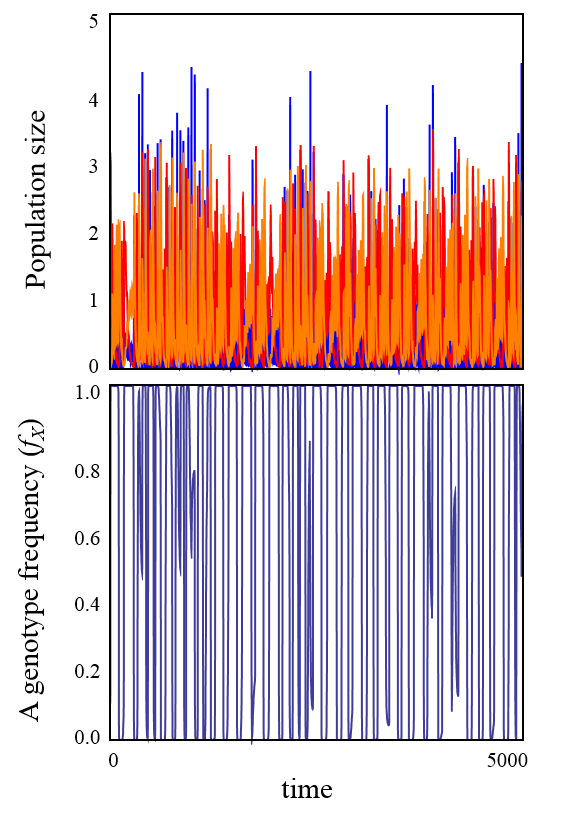

Supplement: S6 Fig — (TIF) [file pone.0227111.s008.tif]
